# Editorial: Atopic dermatitis: from bench to bedside

**DOI:** 10.3389/fmed.2024.1431191

**Published:** 2024-05-29

**Authors:** Jiaman Wang, Baoqing Deng, Yanhua Liang

**Affiliations:** ^1^Department of Dermatology, Cosmetology, and Venereology, Shenzhen Hospital, Southern Medical University, Shenzhen, Guangdong, China; ^2^Department of Dermatology, Shenzhen Baoan Center for Chronic Disease Control, Shenzhen, China

**Keywords:** atopic dermatitis, *Malassezia*, tryptophan, CCR5, Hsp90α

Atopic dermatitis (AD) is a chronic inflammatory, recurrent, life-long, and heterogeneous cutaneous disease characterized by pruritus, dry skin and localized eczema. The pathogenesis of atopic dermatitis has not been fully elucidated due to its complexity. Along with plenty of laboratory-based findings uncovering the pathogenesis of AD, some of the products raised from the laboratory are being widely used in the clinics and achieved encouraging therapeutic efficacy, such as biologics on JAK inhibitors (1).

Huang et al. reviewed the relationship between tryptophan metabolism and the complex network of skin inflammatory response in AD. Tryptophan has long been recognized as a key regulator of immune cells, influencing the occurrence and development of many immune and inflammatory diseases (2). On the basis of analyzing and summarizing a large number of literatures, the authors speculate that exogenous supplementation of tryptophan derivatives, or targeted intervention of gut microbiota (GM), or targeted induction of the Kyn-IDO pathway to accelerate endogenous tryptophan metabolism and produce AhR ligands, may ultimately alleviate clinical symptoms of AD. Now evidences are provided that there is a correlation between tryptophan metabolism and AD immune regulation. However, the biological functions involved in tryptophan metabolism are complex. The causal relationship among tryptophan metabolism, AD and GM as well as the specific molecular mechanisms need to be further determined by laboratory-based research and verified by clinical verification.

Storz et al. analyzed sensitization pattern toward the *Malassezia* spp. and *Candida albicans* among 16 AD patients and 14 healthy controls. The study shows sensitization in general and toward *Malassezia* spp. and *Candida albicans* is increased in AD patients compared to healthy controls, and further, an association between sensitization toward and skin colonization with *Candida* spp., yet an inverse correlation between sensitization toward and colonization with *Malassezia* spp. with increasing disease severity in AD patients. In another study, Chu H et al. reported that the increased sensitization to *Malassezia furfur* was observed in patients with head and neck dermatitis, which is a refractory phenotype of AD. Also, sensitization to *M. furfur* was associated with increased disease severity (3). The results of both studies are consistent, though their sample sizes are very different. It can be concluded that severe AD is associated with increased host sensitivity to mycobiota. More in-depth investigation may forward therapeutic approaches for AD.

Sitko et al. focused on the differences in the detection of circulating Hsp90α between patients with AD and dermatitis herpetiformis (DH). As both are pruritic diseases, AD may be confused clinically with DH. Levels of circulating Hsp90α were determined in serum samples derived from patients with AD (*n* = 31), DH (*n* = 26), contact dermatosis (CD) (*n* = 15), and healthy controls (*n* = 55). Serum levels of Hsp90α were found significantly higher in patients with AD when compared to patients with DH. Though the diagnosis of DH can be made by detecting anti-eTG and anti-tTG autoantibodies, the assessment of Hsp90α levels may help physicians distinguish DH from AD in those cases where serological results regarding the level of anti-eTG and anti-tTG autoantibodies are equivocal with a simultaneous low level of circulating Hsp90α. Standardization of the method to detect Hsp90α and its accuracy, sensitivity, and specificity, is needed before its clinical application.

The study by Zhou et al. shows that CCR5 is identified as the most important key gene associated with both keloid and AD by integrating comprehensive bioinformatics techniques and machine learning methods. The authors used the training datasets of Gene Expression Omnibus and find the overlapping of 449 differentially expressed genes both in keloid and AD. Finally upregulation of CCR5 was validated through two machine learning algorithms of LASSO and SVM-RFE, and confirmed using clinical samples of keloid and AD. CCR5 plays critical roles in the Th cell axis, and provide new insights into the common pathogenesis of these two diseases.

The pathogenesis of AD is very complicated and much remains to be unveiled.

## Author contributions

YL: Conceptualization, Supervision, Writing – review & editing. JW: Writing – original draft. BD: Writing – review & editing.
